# The Challenges of Measuring Informal Care Time: A Review of the Literature

**DOI:** 10.1007/s40273-021-01053-2

**Published:** 2021-07-29

**Authors:** Sean Urwin, Yiu-Shing Lau, Gunn Grande, Matt Sutton

**Affiliations:** 1 Health Organisation, Policy and Economics, School of Health Sciences, University of Manchester, Williamson Building, Oxford Road, Manchester, M13 9PL UK; 2 Division of Nursing, Midwifery and Social Care, University of Manchester, Manchester, UK

## Abstract

Economic evaluations increasingly include the value of informal care, for example, in terms of caregiver health effects or time costs. If an economic evaluation uses caregiving time costs, appropriate measurement of caregiving time is an important first step prior to its valuation. There is no comprehensive overview of the measurement challenges for caregiving time. In this literature review, we searched Medline, Embase, Econlit and Scopus to identify measurement issues and associated studies which reported informal care time that addressed them. The search identified 27 studies that addressed nine measurement issues. There is limited evidence on how to address these issues, although some have received relatively more attention, including incremental time (considered in 16 studies), time measurement method comparisons (six studies) and the inclusion of intangible tasks (four studies). Non-response (considered in only one study) and carer and recipient identification (two studies) were the most wide-reaching measurement concerns, as these determine who is identified as carers. There was no evidence on the consequences of these measurement challenges in terms of impacts on cost-effectiveness ratios and on the total cost of health conditions, which would be a crucial next step. Future research on these issues should consider a range of different settings, as informal care is highly heterogeneous. The measurement of informal care is key for its inclusion in economic evaluations but there is little consensus on how to appropriately measure this type of care.

**Supplementary Information:**

The online version contains supplementary material available at 10.1007/s40273-021-01053-2.

## Key Points

**Table:** 

The issues relating to the measurement of informal care time are important to consider when including informal care time costs as part of an economic evaluation.
The widest reaching issues are those which determine who are identified as carers.
There is a lack of substantial evidence on all measurement problems with caregiving time and most importantly on the consequences of these in terms of cost-effectiveness ratios and the total cost of a health condition.

## Introduction

Informal care is a major component of long-term care for the elderly and those with chronic health conditions of all ages [1, 2]. Given the changing age structure of populations in developed countries, the demand for long-term care is predicted to increase in the future [3]. It is therefore important for research on long-term care to recognise and account for informal carers’ contributions. However, as informal care is a ‘non-market’ good, there are numerous challenges with its definition, measurement and valuation [4–8].

Economic evaluations of a health intervention vary considerably in whether they account for caregiving. These types of analyses aim to inform the optimal allocation of health care budgets [9]. Economic evaluation guidelines in many countries advocate a societal perspective in some form [10–12]. A review of 45 national Health Technology Assessment guidance documents found that 27% of these recommended a societal perspective [13]. The second US panel on cost effectiveness recommended that both a societal and healthcare perspective be used as a reference case with an impact inventory detailing why certain costs were included or excluded [11]. A societal perspective raises the question of whether to account for the spillover effects of an intervention on family members and caregivers. This movement towards a greater consideration of spillovers has contributed to a growing interest in the incorporation of these sorts of spillovers into economic evaluations [14].

Informal care spillovers can be included in a cost-effectiveness ratio in the form of health effects on family members (as the denominator) and as time costs to carers (as the numerator). Two systematic reviews found that the inclusion of informal care time costs in some cases altered the incremental cost-effectiveness ratio of an economic evaluation [15, 16]. Nonetheless, inclusion of these time costs is not the norm [17].

Cost-of-illness analyses are another group of studies where there is a growing interest in the inclusion of informal care as part of a non-healthcare cost. A systematic review of cost-of-illness studies judged that informal care was ‘highly relevant’ for dementia, cancer, mental diseases, multiple sclerosis and stroke, with the exception of arthritis [18], however this study did not consider informal care to children with disabling conditions. If it is both desirable and feasible for a cost-of-illness study to include informal care costs, then it is important to seek comparability.

There are many instruments available to a researcher who wishes to capture informal caregiving time in some form. Examples include the Client Service Receipt Inventory [19], the Caregiver Activity Survey [20], the Caregiver Activity Time Survey [21] and Resource Use Dementia [22]. These instruments are generally used in cases of caregiving to those with dementia, except for the Client Service Receipt Inventory, which has had many variations applied to different conditions. There are now instruments that have been designed to capture caregiving time in palliative care [23–25], kidney disease [26] or cancer settings [27] as well as caregiving to children [28]. Other instruments are not specific to a recipient group but aim to incorporate measurement and valuation into one questionnaire for use as part of an economic evaluation [29, 30].

A possible barrier to more widespread inclusion of informal care time costs in economic evaluation is perhaps the lack of ‘best practice’ guidance [14] and problems with double-counting caregiving as both an outcome (the disutility of caregiving) and a time cost [15, 31]. Recent methods to obtain a ‘pure time cost’ of informal care enable both effects to be included in an economic evaluation [31]. However, this monetary valuation still relies on the use of informal care time. The inclusion of informal care time in economic evaluations requires three stages: definition, measurement and valuation. Valuation has received the most attention, yet measurement is just as crucial. Providing clarity and collating the measurement issues may also help address barriers to the inclusion of the time costs of caregiving for instances where this is appropriate. They may be of further relevance to studies that seek to identify the causal effects of caregiving on, for instance, the health and labour market outcomes of the carer (see Bauer and Sousa-Posa [32] for a review of the literature) or the health and social care utilisation of the recipient [33–36].

Reviews have been undertaken that mention a selection of the measurement challenges when capturing informal care time [4–8], but they have not primarily focused on measurement issues. Instead, they have also focused on the challenges of obtaining a monetary value of informal care and its subsequent inclusion in economic evaluations. Whilst these reviews highlight some of the measurement issues, they do not use a comprehensive search strategy. Therefore, it remains unclear the extent to which measurement challenges with informal care time have been addressed in some form.

In this literature review, we build upon the five previously mentioned review studies [4–8] by being the first to employ a comprehensive literature search strategy to identify studies that have focused on measurement issues of informal care time. For the purpose of this review, we define informal care as unpaid health-related care. The aim of this literature review is to provide a comprehensive overview of the methodological issues with the measurement of informal care time. We approach this aim by (i) identifying studies that addressed methodological issues with the measurement of informal care time, and (ii) mapping these studies to a set of measurement issues.

## Method

Our search used Medline, Embase, Econlit and Scopus (the former three via Ovid databases). We provide the search terms, criteria for inclusion, the resources used for the review as well as the information extracted (with reasons) in Table 1. We chose broad search terms to reflect the variety of alternative terms given to informal care across and within different disciplines. The criteria for inclusion were developed post-hoc as the familiarity of the subject area increased. One author (SU) carried out the search and screening. A second author (YL) screened a 10% sample and resolved any differences in study selection with SU.

**Table 1:** Search strategy and extracted information

**Search terms for the abstract, title or keywords:**
“informal care*” OR “unpaid care*” OR “family care*” OR “lay care*” OR “elder care*”
AND
time OR task* OR activit*
AND
issue* OR bias* OR valid* OR reliab* OR survey* OR challenge* OR method* OR measure* OR questionnaire* OR instrument*
**Criteria for inclusion:**
English language
Peer reviewed
Empirical study that reported data on the hours or minutes of care
Applies to informal care where the providers are adults
Addressed measurement issues regarding informal care time
**Resources:**
Ovid
Econlit
Embase
Medline
Scopus
Web of Science
Forward and backward search of review articles
**Information extracted (with reasons) to be presented in Table** 3:
Year of publication (how the literature has developed over time)
Country of origin of the data used (cultural and institutional differences surrounding the provision and receipt informal care)
Health condition of the care recipients (care recipients are a heterogeneous group)
Method of time measurement (first consideration of a researcher when capturing informal care information)
Sample size (external validity of the study)

*The full search syntax used for Ovid is available in Online Appendix Table A1, see electronic supplementary material (ESM)

The inclusion criteria aimed to focus on studies that methodologically addressed issues in informal care time assessment, rather than mentioning the possibility of one. We included only peer-reviewed studies, which acted as a filter for quality. We only included empirical studies, as these would capture informal care time, which is of use to economic evaluations that incorporate time costs. Studies centred on caregivers who are children were not included because the measurement concerns associated with capturing and valuing the time use of children are very different to adults [37].

We also checked the reference lists and citations (up to January 2020) of the five review studies [4–8]. We chose these review studies based on knowledge of the area by the authors. We performed this search using the built-in features available in Web of Science. This additional search acted as a check for the main literature database search. Further studies were included based on the knowledge of the authors if not already identified in either of the searches.

**Fig. 1:**
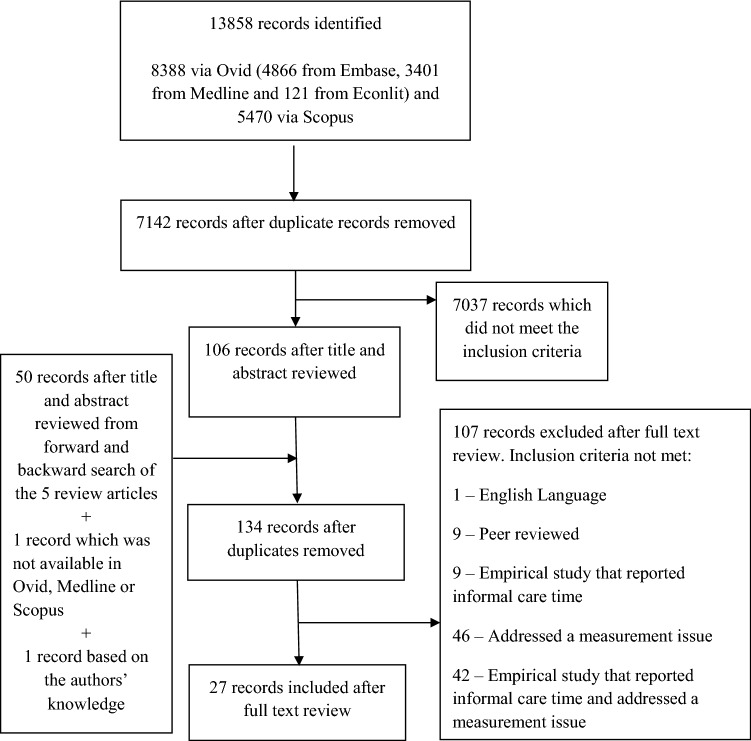
Article selection process

We obtained 13,858 records for a search to December 2018 with no date restrictions from Econlit, Embase, Medline and Scopus databases, which reduced to 7142 after de-duplication. After screening the title and abstract of the remaining records in Endnote, 106 studies met the inclusion criteria. A 10% check by YL identified the same papers as the initial screen plus four extra papers that were excluded after discussion. The addition of a further 50 studies from the review studies search and one from the authors’ knowledge, as all the journal articles were not available via Ovid or Scopus, resulted in 134 studies for full-text review (after again removing duplicated records). Based on a full-text review of these studies, focusing on the empirical content, we decided amongst all authors whether they addressed a measurement issue. We used the review studies as a starting point for already identified measurement issues. In total, we identified a final 27 studies (Fig. 1). Of these, one study was from a journal not available in the literature databases and another had no abstract, which substantially decreased the likelihood of identification from the literature search.

The decision on what information to extract (shown in Table 1) and present as a summary outline of the included studies was discussed with all authors. Information extraction was performed by the lead author (SU). We further summarised the directional impact the measurement issues had on informal care time, and the methods to address these issues.

## Results

### Identified Measurement Issues

We identified nine distinct measurement issues considered in 27 studies from full-text review [22, 23, 28, 29, 38–60]. These are defined and described in Table 2. The most frequently addressed measurement issue was incremental time (*n* = 16) [23, 28, 29, 38, 40, 43, 45–50, 53, 54, 56, 58] followed by those that addressed the time measurement method (*n* = 6) [28, 39, 41, 57, 58, 60].

**Table 2:** The identified measurement issues

Measurement issue	Definition	Number of studies
Incremental informal care time	(i) The amount of total caregiving time that is due to the needs of a particular health condition of a recipient. (ii) The amount of time that is classified as caregiving	16 [23, 28, 29, 38, 40, 43, 45–50, 53, 54, 56, 58]
Joint production	The performing of multiple tasks at the same time	3 [42, 58, 60]
Time-bound tasks	The degree to which certain caregiving tasks have to be performed at the same time each day	1 [42]
Time measurement method	Different means of collecting informal time care information (e.g. diary vs recall methods)	6 [28, 39, 41, 57, 58, 60]
Intangible tasks	Tasks that have no tangible end product such as social and emotional care	4 [22, 44, 59, 60]
Carer and recipient identification	Informal care information compared between carers or recipients within the same dyad, or from one perspective across different measurement methods	2 [39, 55]
Multiple caregivers	Informal care provided by someone who is not the primary caregiver	2 [50, 51]
Aggregation of informal care tasks	Means of collecting informal care information through list-based tasks or one aggregated question	1 [58]
Non-response bias	Carers may be more or less likely to take part in surveys (unit non-response) or answer caregiving questions (item non-response) than non-carers	1 [52]

The largest number of studies were based on carers/recipients residing in the USA (*n* = 5) [43, 45–47, 49], followed by the Netherlands (*n* = 4) [29, 42, 52, 58], shown in Table 3. Included studies most often sampled care recipients with dementia (*n* = 11) [22, 44, 46, 48–51, 53, 54, 59, 60], recipients who had suffered a stroke (*n* = 5) [29, 40, 43, 45, 56] and recipients without a specified health condition (*n* = 5) [39, 42, 52, 55, 58]. The recall method to obtain caregiving information was used in all but two studies, which either used the time diary method [42] or did not specify the method [22]. Only six studies addressed more than one measurement issue [28, 39, 42, 50, 58, 60]. Sample sizes were typically under 1000 carers or recipients. Table 4 presents a summary of the directional impact of not addressing informal care time issues and the methods used to address them. The next section provides further detail of the studies within each measurement issue.

**Table 3:** Characteristics of each included study (alphabetical order)

Study	Country	Care recipient group	Method of measurement	Sample size	Measurement issue
Bittman et al. [39]	Australia	Not specified	Recall questionnaire and time diary	918 carers via recall and 889 in time use survey	(1) Carer and recipient identification (2) Time measurement method
Dewey et al. [40]	Australia	Stroke	Recall questionnaire	340 care recipients	(1) Incremental time
Dumont et al. [23]	Canada	Palliative care patients	Recall questionnaire	268 caregivers; 248 recipients	(1) Incremental time
Flyckt et al. [41]	Sweden	Psychosis	Recall questionnaire and time diary	118 caregivers; 107 recipients	(1) Time measurement method
Hanly et al. [38]	Ireland	Cancer (head and neck)	Recall questionnaire	180 caregivers; 180 recipients	(1) Incremental time
Hassink and Van den Berg [42]	Netherlands	Not specified	Time diary	199 caregivers	(1) Joint production (2) Time-bound tasks
Hickenbottom et al. [43]	USA	Stroke	Recall questionnaire	6784 individuals with no stroke, 281 stroke without health problem, 375 stroke with health problem (Asset and Health Dynamics Study)	(1) Incremental time
Jakobsen et al. [44]	Denmark	Dementia	Recall questionnaire	469 caregivers	(1) Intangible tasks
Jarbrink et al. [28]	UK	Autism spectrum disorder	Recall questionnaire and time diary	15 carers (parents) and 17 recipients (children)	(1) Incremental time (2) Time measurement method
Joo et al. [45]	USA	Stroke	Recall questionnaire	8525 respondents from the Health and Retirement Study; 230 had a stroke and 8295 were the non-stroke group	(1) Incremental time
Langa et al. [46]	USA	Dementia	Recall questionnaire	7443 respondents to the Asset and Health Dynamics Study	(1) Incremental time
Langa et al. [47]	USA	Lung disease	Recall questionnaire	7443 respondents to the Asset and Health Dynamics Study	(1) Incremental time
Leicht et al. [48]	Germany	Dementia	Recall questionnaire	173 dementia patients; 176 matched controls	(1) Incremental time
Moore et al. [49]	USA	Dementia	Recall questionnaire	2043 caregivers	(1) Incremental time
Neubauer et al. [50]	Germany	Dementia	Recall questionnaire (RUD)	357 caregivers (26 of these were not the primary caregiver) to someone with dementia	(1) Multiple caregivers (2) Incremental time
Neubauer et al. [51]	Germany	Dementia	Recall questionnaire (RUD)	357 caregivers; 390 patients	(1) Multiple caregivers
Oldenkamp et al. [52]	Netherlands	Not specified	Recall questionnaire	8443 caregivers as part of the LifeLine study, 2002 caregivers who gave consent for the informal care questionnaire and 965 returned the questionnaire	(1) Non-response bias
Ostbye and Crosse [53]	Canada	Dementia	Recall questionnaire	N/A	(1) Incremental time
Paraponaris and Davin [54]	France	Dementia	Recall questionnaire	513 caregivers to someone with dementia matched to 4167 individuals	(1) Incremental time
Rutherford and Bu [55]	England	Not specified	Recall questionnaire	521 spousal caregivers; 799 spousal care recipients	(1) Carer and recipient identification
Skolarus et al. [56]	USA	Stroke	Recall questionnaire	892 stroke survivors matched to 892 individuals without a stroke	(1) Incremental time
Timonet-Andreu et al. [57]	Spain	Heart failure	Recall questionnaire (perceived and actual)	478 family caregiver dyads	(1) Time measurement method
Van den Berg and Spauwen [58]	Netherlands	Not specified	Recall questionnaire and time diary	199 caregivers	(1) Aggregation of informal care tasks (2) Incremental time (3) Joint production bias (4) Time measurement method
Van den Berg et al. [29]	Netherlands	Rheumatoid arthritis and stroke	Recall questionnaire and time diary	365 caregivers (*n* = 147 to those with RA; *n* = 218 to those with stroke)	(1) Incremental time
Wimo et al. [22]	Not reported	Dementia	Not reported	15 caregivers	(1) Intangible tasks
Wimo et al. [59]	Sweden	Dementia	Recall questionnaire (RUD)	92 caregivers; 92 recipients	(1) Intangible tasks
Wimo et al. [60]	Sweden	Dementia	Recall questionnaire (RUD) and direct observation	14 caregiving dyads	(1) Intangible tasks (2) Joint production bias (3) Time measurement method

*RA* rheumatoid arthritis, *RUD* Resource Utilization in Dementia instrument

**Table 4:** Details on the challenges, direction on caregiving hours and methods to address each issue

Measurement issue	Detail on the challenge this issue presents	Impact on the direction of time reports from not addressing the issue as reported by the studies	Methods in reviewed studies to address the issues
Incremental care time	Whether to adjust total caregiving hours for those attributable to the health conditions of a recipient	Overestimation	Ask carers to explicitly report time due to the needs of the recipient’s health condition [40, 50] Ask carers to report care time before and after the onset of the recipient’s health condition [23] Between-subject comparison using regression adjustment and/or matching methods [43, 46, 47, 49, 53, 56] Difference-in-difference with propensity score matching [45]
	Whether to adjust reported caregiving for normal and caregiving-related time	No difference	Stratify carers between co- and extra-residential carers as evidence this is an issue [38] Comparison of reported household tasks with a time diary (between-subject comparison) [29]
Joint production	Whether to adjust hours for joint production	Overestimation	Adjust reported time from a time diary [58]
Time-bound tasks	Whether to obtain information on types of tasks		
Time measurement method	Whether to collect direct observation, recall or time diary	Mixed (recall vs time diary)	Comparisons within individuals or across surveys between the recall method and the time diary method [28, 39, 41, 58, 60]
Intangible tasks	Whether to collect information on intangible tasks	Underestimation	Include intangible tasks in the questionnaire or time diary [22, 44, 59, 60]
	Whether to address large hourly reports	Overestimation	Subtract time from intangible tasks to allow the total caregiving hours not to exceed 168 h in a week [22, 44, 60]
Carer and recipient identification	Whether to collect information from the provider or recipient	Provider reported time greater than recipient reported time	Compare provider and recipient declarations of caregiving [55]
Multiple caregivers	Whether to collect information of further caregivers	Underestimation of total care time to a recipient	Obtain information from a non-primary caregiver [50, 51]
	Whether to use the primary or non-primary caregiver to collect information on all caregivers		
Aggregation of informal care tasks	Whether to collect a global or task-based measure of caregiving time	Total time using one aggregated tasks question underestimated relative to separate tasks	Compare aggregated and separated tasks questions [58]
Non-response bias	Whether certain groups of carers are less likely to take part in surveys	Overestimation	

### Measurement Issues

#### Incremental Informal Care Time

Incremental informal care time relates to either (i) the amount of total caregiving time that is due to the needs of a particular health condition of a recipient or (ii) the amount of caregiving time excluding the day-to-day tasks the provider would have done anyway had they not become a carer. We offer these two definitions because the caregiving time remaining after adjustment for (ii) could still be due to several recipients or to one recipient who has several health conditions.

Studies under this issue have focused on methods that attribute a part of total informal care time to the health condition needs of a recipient. These studies have used between-subject comparisons by comparing caregiving to those with and without a specific health condition through regression adjustment and/or matching methods [43, 46, 47, 49, 53, 56]. A further study used difference-in-difference methods that compared total caregiving before and after the onset of a stroke with a propensity-score-matched control group over the same time period [45]. These studies have focused on informal care time attributable to dementia or stroke.

Some studies explicitly asked carers to only report tasks they would not have performed prior to the onset of their recipients stroke [40] or dementia [50]. A similar means of addressing this is to ask respondents to report household tasks prior to caregiving and during caregiving to calculate the difference [23].

However, it is not clear whether and by how much total reported caregiving time is ‘normal’ or what are ‘caregiving’-related tasks given that a starting point of a caregiving role may not be clear. A focus has been on correcting reported household tasks or instrumental activities of daily living tasks. A within-subject comparison method calculated the difference between caregiving from the recall method and time diary, where the diary explicitly asked carers to report time on tasks for themselves and for their recipient [58]. A between-subject comparison used time diary data for household tasks from a general population to compare with carer-collected household task information [29]. Another means of identifying this issue is to perform analysis of hours on separate sub-groups of carers. For instance, by whether the carer and recipient co-reside, under the assumption that carers who co-reside with their recipient are less able to distinguish between normal and caregiving tasks [38].

#### Joint Production

Performing more than one task at a time may be more common among carers compared with non-carers or high-intensity carers compared with low-intensity carers. Joint production is thus a characteristic of a carers’ time use and may be a source of variation among carers who appear to provide a similar amount of care. It would therefore be important to understand if and by how much carers combine tasks.

Joint production was captured with time diaries for the three studies that addressed this issue [42, 58, 60]. Two of the three studies identified what type of tasks were jointly produced and which groups of carers were likely to jointly produce [42, 60]. Hassink and Van den Berg [42] showed that informal care was most commonly combined with household activities than leisure or paid work using Dutch data. Wimo et al. [60] showed that 85% of instrumental activities of daily living were jointly produced with other tasks. Unemployed caregivers were a sub-group of carers that jointly produced more than employed carers as paid work was harder to combine with caregiving tasks [42].

Only one study that considered joint production attempted to adjust for this issue [58]. They used a modified time diary that was adjusted to subtract an amount of time from each task depending on the number of tasks performed in a fixed period. This resulted in a 36% reduction in care provision time from 546.37 to 348.91 min per week, on average [58].

#### Time-Bound Tasks

The degree to which informal care-related tasks can be shifted within and between days has implications on the opportunity cost of caregiving. For example, if a carer has to perform personal care tasks at certain times of the day, this will entail a higher opportunity cost than other tasks that could be performed at different times such as household tasks. It would therefore be appropriate to account for the different types of tasks when measuring and valuing informal care.

The one study that considered this issue explored whether, and what type of caregiving tasks were time-bound [42]. Time diary data enabled analysis that looked at the timing of activities throughout the course of a day. Hassink and van den Berg [42] through a comparison of working and non-working carers identified that personal care tasks were not shifted throughout the course of the day. Their results indicated an added opportunity cost of these types of caregiving tasks, a further consideration with the measurement of informal care when assessing the labour market implications of caregiving.

#### Time Measurement Method

Alternative methods to capture informal care time may yield very different reports of time. This raises the concern of comparability between time measurement methods, and whether the conclusions of studies are partly due to the method used.

Studies that addressed this issue were mainly concerned with differences in the reported hours between each method of collecting informal care time information. These have involved comparisons of the recall method with either a time diary [28, 39, 41, 58, 60], direct observation [60] or a different recall method version [57]. These studies assumed that the time diary, a means of direct observation or a particular version of the recall method, was the gold standard. A further distinction was that all studies used two methods applied to the same group of carers [28, 41, 57, 58, 60], except for one study that used two different groups of carers [39].

The recall method had lower reported caregiving time compared with the diary for two studies, where the first found a difference of 55 min per day (equivalent to 6.41 h per week) [58] (with a diary unadjusted for joint production) and the other a difference ranging from 2 to 20 h per week [41]. A further study found the recall method had larger reports of caregiving time by 17 hours per week relative to a time diary among informal carers although only nine of the 15 respondents provided recall information [28]. Once the time diary was corrected for joint production in Van den Berg and Spauwen [58], the recall method reported higher caregiving time by 2 h per day (equivalent to 14 h per week) driven by the difference in household daily living tasks and instrumental activities of daily living tasks. A further study with Australian data found the recall method produced higher reports of time compared with the diary. However, these two methods were applied to different samples of carers, which may be reflective of the unreported differences in the characteristics of both carer groups [39].

#### Intangible Tasks

The majority of informal care tasks have a tangible end product. For example, when a carer cooks for their recipient, this results in a consumable meal. However, some tasks have no end product, such as being on-call for a care recipient. Carers are likely to be on call for most of the day, which would therefore account for a large part of total caregiving hours. The challenge is two-fold: (i) to understand what sort of intangible tasks to count as caregiving and (ii) to understand how to address reports of supervision/on-call tasks that are greater than the hours in a day/week, if at all.

The four studies that addressed intangible tasks were all focused on care of someone with dementia [22, 44, 59, 60]. They recognised that supervision, on-call and prevention-type tasks were likely to be more common in those who cared for someone with this condition.

One study addressed intangible tasks by demonstrating that supervision tasks accounted for 151 (50.5%) out of 299 h of caregiving time across a month and, using regression analysis, that these hours were larger for co-residing carers than non-co-residing carers [59].

The remaining studies attempted to address the possible overestimation of supervision time from its inclusion in time instruments. This was through the addition of extra questions on sleep and the amount of time a dementia patient is left alone to help the carer adjust their estimates of caregiving [60], or to cap hours at the maximum time available by first subtracting hours from supervision tasks followed by other tasks [22, 44].

#### Carer and Recipient Identification

Informal caregiving involves both a provider and a recipient. This offers two perspectives from which to collect caregiving time information. If providers are less likely to make claims of caregiving than recipients, then comparability issues would arise across studies dependent on the perspective used; for example, if recipients report different caregiving hours than caregivers within the same dyad. This issue can also include identification of a provider from two methods: a declaration question with the recall method or from recorded activity with a time diary.

Two studies explored the degree to which this issue occurred, one that compared discrepancy between provider and recipient reports [55] and another that compared the identification of providers between a diary and recall questionnaire [39]. The former considered spouses over 50 years old from the English Longitudinal Study of Ageing and found that among 799 reports of care receipt, only 420 (52.6%) of these claims were confirmed by the nominated provider. They also found that carers reported providing more hours relative to their recipient. The latter study used a diary and recall questionnaire in the Australian 1997 Time Use Survey. They found that 240 carers were identified as such in the time diary but not in the recall questionnaire (referred to as ‘non-identified carers’) [39].

#### Multiple Caregivers

Those in need of assistance with daily activities can receive care informally from multiple providers. This issue relates to whether it is necessary to account for all caregivers, which can be challenging if not all carers co-reside with their recipient/s. Further, if non-primary carers allocate substantial amounts of time to caregiving responsibilities, then the cost of obtaining this information would need to be weighed against the expected outcome of including this group in subsequent analysis.

Two studies identified to what degree the exclusion of caregiving information from the non-primary caregiver resulted in an underestimation of care time [50, 51]. Both studies were undertaken in a dementia setting and applied the resource use dementia questionnaire from the same sample of dementia patients and carers, but asked the respondent to record all caregiving time received by the same person. They found that more than half of the carers in their samples reported additional carers [50, 51]. Inclusion of just the hours of the primary carer resulted in a 14% underestimation in the total hours of care provided to a particular care recipient [50].

Neubauer et al. [51] took this issue further and showed that total informal care time provided to a particular recipient as reported by the non-primary caregiver had only a small difference of 1.3 hours less per day compared with interviewed primary caregivers, with differences driven by differences in supervision time. Although, these differences were only among 24 non-primary caregivers.

Therefore, there is some evidence that the exclusion of non-primary carers will result in an underestimation of total caregiving hours. However, all studies on this issue that are included in this review have been undertaken in a dementia setting. It is not clear because of small sample sizes whether obtaining information about total caregiving provision from different caregivers would result in substantially different estimations of hours.

#### Aggregation of Informal Care Tasks

There are many methods to obtain informal care time information. One issue is whether to ask one aggregated question or to sum hours from separate questions on different informal care tasks. If there were differences between the two approaches, this would limit comparability across studies that use the two methods.

The study that considered this issue sought to identify whether carers answered differently depending on the structure of the question. Carers reported spending less time on household tasks via an aggregated approach than via separate tasks. Both approaches used the recall method and provided some evidence that one aggregated question on informal care time may result in an underestimation of caregiving [58].

#### Non-Response Bias

Non-response rates may be particularly high in caregiver samples. If non-response is related to certain characteristics, then particular groups of caregivers may be under (or over)-represented in a carer sample. This would present important challenges regarding the representativeness and collection of carer information.

Only one study aimed to identify if non-response was an issue within a carer sample [52]. They explored whether non-response and the separate but related construct of non-consent was related to carer characteristics using a large population-based cohort study in the Netherlands [52]. Oldenkamp et al. [52] identified carers from the cohort study and asked if they would consent to a further questionnaire. Responders were more likely to have provided 4–8 h and > 8 h of household, personal and other care tasks compared with non-responders. The results were similar for consenters compared with non-consenters. These results fit with the hypothesis provided by Oldenkamp et al. [52] that carers are more likely to participate where a research topic is relevant to their everyday life.

## Discussion

### Main Findings

A complete understanding of the challenges in measuring informal care time is key if this aspect of informal care is to be more widely included in economic evaluations. We conducted a comprehensive review of the literature that addresses methodological issues in the assessment of informal care time. This literature is a subset of studies centred on the assessment of informal care time. We identified nine issues in the measurement of informal care time. We found limited evidence that addressed these issues, as only 27 studies were identified. Incremental informal care time was the most commonly addressed issue, followed by the time measurement method and intangible task issues.

The impact these challenges would have on reported hours was clear in some cases. Based on the studies identified in this review, not accounting for incremental informal care time (under the first part of the issue) due to the health condition of a recipient resulted in an overestimation of hours. In contrast, the exclusion of intangible tasks, additional non-primary carers and the aggregation of informal care tasks (compared with separate tasks) resulted in an underestimation of hours. Out of these four challenges, intangible tasks arguably had the greatest impact on total reported caregiving compared with the other challenges. This was in cases of caregiving to those with dementia, given that it made up a large percentage of the total reported hours [59]. The issue of intangible tasks will be less of a concern among conditions that do not require substantial supervision or time input in general. Given that many of the studies apply to dementia, the methods used for incremental informal care time and joint production are likely to be the same for other conditions. However, it may be more difficult, if asked directly, for carers to separate between caregiving time and usual non-caregiving time with conditions such as dementia compared with diabetes or a stroke, where a starting point for the provision of informal care is clearer.

The impact on caregiving time was not as clear for joint production adjustment, time measurement methods and whether carers could separate between normal and caregiving tasks (under the second part of the incremental time issue). The limited evidence from the first issue suggested that not accounting for joint production resulted in an overestimation of time, but this was only identified via one study with a time diary. Other studies that addressed the issue of joint production focused more on identifying that joint production was commonplace among carers. For time measurement methods, the evidence was mixed as the recall method was either an overestimate relative to the diary or, if the diary was adjusted for joint production, the recall method became an underestimate. There was mixed evidence regarding whether carers were able to distinguish between normal and caregiving tasks as some studies found no evidence that this was an issue, whereas one found that carers could not distinguish between these types of tasks. A further study found carers reported less household cleaning hours than equivalent time use from a population-based time use survey.

The most wide-reaching issues were carer and recipient identification, non-response bias and the issue of multiple carers, as these related to problems in categorising and obtaining information from carers. Consequently, these measurement concerns would have a knock-on effect on all the other issues that directly related to the measurement of time. For example, recipient reports may identify previously under-reported carers. Therefore, if the amount of time reported by recipients was different to that reported by carers, then methods to adjust for incremental informal care time and joint production will be impacted. It was not clear to what extent the correction for incremental caregiving time and joint production would have resulted in some carers having zero or very little reported hours of care and thus being classified as non-carers. The issue of incremental caregiving time is also one of the most important issues because only the incremental time should be included if economic evaluations wish to include time costs.

### Applicability of the Measurement Issues

#### Monetary Valuation Methods

The applicability of each measurement issue depends on how economic evaluations incorporate informal care. The most widely used monetary valuation methods for informal care in cost-effectiveness and cost-of-illness analyses are those that only consider time costs—the proxy good and opportunity cost methods [18]. The proxy good method is implemented by assigning one wage rate to all informal care time or different wage rates to certain task types. All measurement issues are relevant with this approach. However, the use of one aggregated question, which would only be necessary if one wage rate is used, may result in lower total hours than questions for separate tasks. However, the evidence on this was limited as it was based on only one study [58].

The opportunity cost can also be implemented in various way by asking carers to consider forgone paid work, unpaid work and leisure (or combinations of these), which are then multiplied by their respective prices. These versions rely implicitly on the carer to make a judgment of the number of hours spent caregiving and what was given up in order to provide this care [29]. Similarly, the time use of carers can be compared with non-carers through matching and/or regression adjustment. Time measurement issues would still affect the opportunity cost, but could not be addressed unless caregiving time was collected. This would be the case in instances where lost work hours are valued using a regression approach with a comparison group. Other versions collect informal care time assuming just paid work is forgone and value carers time at their market wage rate or some other wage rate [15]. Under this version, more issues can be addressed as caregiving time is collected. Regardless of the opportunity cost approach taken, it would be desirable to collect caregiving time to assess how much time is displaced. In particular, collection of separate tasks would provide extra detail.

Other monetary valuation methods such as the wellbeing method [61, 62] and willingness to pay/willingness to accept methods [63–66] cannot be combined with carer time costs in the numerator of the cost-effectiveness ratio [7]. The wellbeing method uses informal care time in the derivation of a monetary value. Both the wellbeing methods and willingness to pay/accept derived monetary valuation can be multiplied by informal care time collected in an economic evaluation. Therefore, the measurement issues would still affect these monetary valuation methods in the same way as the proxy good method.

#### Non-Monetary Valuation Methods

In terms of non-monetary valuation methods, health effects are recommended to be included as quality-adjusted life-years (QALYs) on the effect side of the cost-effectiveness ratio [7]. As these are not directly related to time measurement, issues such as joint production do not affect this valuation method. However, issues regarding identification such as carer and recipient identification, non-response and the inclusion of multiple carers become the relevant measurement issues. For example, if economic evaluations use recipient information to identify carers to obtain health effects, recipients may under-report whether they have a provider.

Recent developments have been made to obtain ‘pure time’ costs of caregiving via conjoint analysis with a discrete choice experiment that adjusts for other effects of caregiving such as health [31]. As a result, this avoids double-counting the health effects of caregiving, which thus allows both time costs and health effects to be included in the cost-effectiveness ratio. This would also mean a greater exposure to informal care measurement issues in both the numerator and denominator of the cost-effectiveness ratio.

#### Secondary Data

The nine issues we have identified are important for analysis beyond economic evaluations, in particular, in studies that use secondary data to consider the causal effects of informal care on work, wages and health of the provider or healthcare use of the recipient [67]. For these studies that use secondary data, the researcher is constrained by what information is already collected. For instance, some surveys may not explicitly capture intangible tasks.

Across secondary datasets, a key distinction is between population-based time use surveys (such as the Multinational Time Use Survey) and household-based panel surveys (such as the Health and Retirement Study in the US and the UK Household Longitudinal Study). Of importance with these surveys is the separation between normal and caregiving tasks as opposed to the proportion of caregiving time due to a health condition of a recipient. Studies assessing this issue have used small-scale surveys [29, 58], where generalisability is a concern.

Some issues may not be of major importance in an economic evaluation but serve more of a purpose in secondary data analysis. For example, differentiating between time-bound and non-time-bound caregiving tasks would be of relevance in understanding the heterogenous effects of caregiving. More specifically, carers who must perform certain caregiving tasks at particular times of the day may have different health and labour market effects than those who do not perform time-bound tasks. To accommodate this, collection of informal care time across a list of activities would thus be recommended as opposed to the collection of one aggregated question [68].

### Gaps in the Literature and Future Directions

A deeper understanding of the relevance of measurement issues to the valuation methods involves knowledge of how these measurement issues are inter-related. For example, comparisons of the recall method and the time diary rely upon whether the time diary is adjusted for joint production. Means to cap large reports of intangible tasks would extend to joint production issues as capping hours may result in excluding hours that were jointly produced.

There are numerous issues with the measurement of any form of time; some of which were not identified in this review. These include the wording of the question, the question order in the survey, the recall time period, justification bias and whether caregiving was provided within or outside of the household. We found no studies that addressed these issues explicitly, although it would have been possible for some of these issues to have operated through the measurement issues that were considered. For example, incremental caregiving time would have been affected by whether caregiving was provided inside or outside the household. Co-residing carers may have been less able to recall the starting point at which they became a carer and therefore to separate normal and caregiving tasks [38].

For the majority of measurement issues, the studies that addressed them only identified the degree to which they occurred and few corrected for the issues. It would be beneficial for future research to explore the consequences of each issue, in terms of the materiality of their impact on the cost effectiveness of an intervention. This may be through sensitivity analysis, for example by assessing the impact of correction for incremental caregiving time, use of an aggregated or list-based caregiving question, or methods to cap intangible tasks. Exploration of this would represent an extension of the work that has looked at whether the inclusion of informal care affects the cost effectiveness of interventions [15, 16].

It has been posited that in the future, off-the-shelf estimates of informal care time costs or health effects could be produced [4], which would reduce the resources required for conducting a cost-effectiveness and cost-of-illness analysis. Grosse et al. [4] further state that these estimates would vary according to the measurement and valuation of informal care time. Specifically, in terms of measurement, it would be necessary to first show that patterns of these measurement problems are stable across different settings and then provide correction factors for each measurement issue including incremental caregiving time, joint production and time measurement methods.

### Limitations of the Review

Judgement of whether a study addressed a measurement issue was based on assessment of the literature by the lead author. Whilst the choice of studies to include was to some degree subjective, the use of review articles through a reference list and citation search helped provide a check for the coverage of the literature search. This judgment of the lead author was calibrated against a second reviewer (YL), who performed a 10% title and abstract screen of the de-duplicated records. We are confident that we have identified a substantial number of all measurement issues. We may not have identified all the studies that encompassed a measurement issue, in particular with condition-specific incremental informal care time such as paediatric care, because they did not consider time measurement issues in detail. Nonetheless, the methods and implications are likely to be similar for studies under this issue. Also, there may be studies published after our search dates that methodologically address measurement issues. It is likely that studies outside the search dates will cite those identified in our review; therefore, our review serves as a starting point for research focused on informal care time. A further limitation was that only the lead author carried out the screening and eligibility assessment of all of the studies. However, throughout the process, numerous meetings of all authors were held in order to clarify the criteria for each stage of the review.

This review was limited to quantitative articles that were peer-reviewed and written in English. Even so, our search produced over 7000 studies. Inclusion of grey literature would have substantially increased the number of studies and prohibited the feasibility of carrying out the review. Similarly, we did not include qualitative studies. These types of studies would have complemented the issues identified in our review and helped identify more. It may be useful for a qualitative version of this review that utilises studies of carer and expert interviews to provide further evidence for this research question.

## Conclusion

Informal care is a non-market good with little means of verification compared with formal means of care or market work. It therefore requires a detailed understanding of issues relating to its measurement. We show that there is much uncertainty regarding what informal care-related questions are fundamentally capturing due to the range of issues we have identified and the scope for future work. Incremental caregiving time has received the most attention in the literature, whereas other issues have received little. This review has demonstrated that if informal care is to be more widely included in economic evaluations, then researchers working with caregiving information need to be aware of the challenges we have identified and understand the likely implications they can have on the research objective.

## Electronic supplementary material

Below is the link to the electronic supplementary material.

Supplementary file1 (DOCX 13 kb)
