# Supplementary material for: The Challenges of Measuring Informal Care Time: A Review of the Literature
Source: Pharmacoeconomics. 2021 Jul 29;39(11):1209–23. doi: 10.1007/s40273-021-01053-2 (PMC8516777; doi:10.1007/s40273-021-01053-2)
Supplement: Supplementary file 1 — Supplementary file1 (DOCX 13 kb) [file 40273_2021_1053_MOESM1_ESM.docx]

# Appendix

Table A1: Ovid search syntax

(<informal care>* or <unpaid care>* or <family care>* or <lay care>* or <elder care>*).ti. or (<informal care>* or <unpaid care>* or <family care>* or <lay care>* or <elder care>*).ab. or (<informal care>* or <unpaid care>* or <family care>* or <lay care>* or <elder care>*).kw.

(time or <task>* or <activit>*).ti. or (time or <task>* or <activit>*).ab. or (time or <task>* or <activit>*).kw.

(<issue>* or <bias>* or <valid>* or <reliab>* or <survey>* or <challenge>* or <method>* or <measure>* or <questionnaire>* or <instrument>*).ti. or (<issue>* or <bias>* or <valid>* or <reliab>* or <survey>* or <challenge>* or <method>* or <measure>* or <questionnaire>* or <instrument>*).ab. or (<issue>* or <bias>* or <valid>* or <reliab>* or <survey>* or <challenge>* or <method>* or <measure>* or <questionnaire>* or <instrument>*).kw.
